# Beyond Systemic Lupus Erythematosus and Anti-Phospholipid Syndrome: The Relevance of Complement From Pathogenesis to Pregnancy Outcome in Other Systemic Rheumatologic Diseases

**DOI:** 10.3389/fphar.2022.841785

**Published:** 2022-02-15

**Authors:** Silvia Cavalli, Paola Adele Lonati, Maria Gerosa, Roberto Caporali, Rolando Cimaz, Cecilia Beatrice Chighizola

**Affiliations:** ^1^ Department of Clinical Sciences and Community Health, Research Center for Adult and Pediatric Rheumatic Diseases, University of Milan, Milan, Italy; ^2^ Clinical Rheumatology Unit, ASST G. Pini & CTO, Milan, Italy; ^3^ Experimental Laboratory of Immunorheumatological Researches, IRCCS Istituto Auxologico Italiano, Cusano Milanino, Italy; ^4^ Pediatric Rheumatology Unit, ASST G. Pini & CTO, Milan, Italy

**Keywords:** rheumatology, pregnancy, complement, biomarker, pathogenesis

## Abstract

Evidence about the relevance of the complement system, a highly conserved constituent of the innate immunity response that orchestrates the elimination of pathogens and the inflammatory processes, has been recently accumulated in many different rheumatologic conditions. In rheumatoid arthritis, complement, mainly the classical pathway, contributes to tissue damage especially in seropositive subjects, with complement activation occurring in the joint. Data about complement pathways in psoriatic arthritis are dated and poorly consistent; among patients with Sjögren syndrome, hypocomplementemia exerts a prognostic role, identifying patients at risk of extra-glandular manifestations. Hints about complement involvement in systemic sclerosis have been recently raised, following the evidence of complement deposition in affected skin and in renal samples from patients with scleroderma renal crisis. In vasculitides, complement plays a dual role: on one hand, stimulation of neutrophils with anti-neutrophil cytoplasmic antibodies (ANCA) results in the activation of the alternative pathway, on the other, C5a induces translocation of ANCA antigens, favouring the detrimental role of antibodies. Complement deposition in the kidneys identifies patients with more aggressive renal disease; patients with active disease display low serum levels of C3 and C4. Even though in dermatomyositis sC5b-9 deposits are invariably present in affected muscles, data on C3 and C4 fluctuation during disease course are scarce. C3 and C1q serum levels have been explored as potential markers of disease activity in Takayasu arteritis, whereas data in Behçet disease are limited to *in vitro* observations. Pregnancies in women with rheumatologic conditions are still burdened by a higher rate of pregnancy complications, thus the early identification of women at risk would be invaluable. A fine-tuning of complement activation is required from a physiological progression of pregnancy, from pre-implantation stages, through placentation to labour. Complement deregulation has been implicated in several pregnancy complications, such as recurrent abortion, eclampsia and premature birth; low complement levels have been shown to reliably identify women at risk of complications. Given its physiologic role in orchestrating pregnancy progression and its involvement as pathogenic effector in several rheumatologic conditions, complement system is an attractive candidate biomarker to stratify the obstetric risk among women with rheumatologic conditions.

## Introduction

The management of pregnancy has been revolutionized by the introduction of non-invasive prenatal screening tests, which allow assessing the risk of hypertensive complications and placental insufficiency. Nevertheless, pregnant women with rheumatologic conditions still experience a higher rate of pregnancy morbidity, mainly in terms of intra uterine growth restriction (IUGR), pre-eclampsia, eclampsia and preterm delivery. This occurs despite the fact that, in the recent years, awareness about reproductive issues in rheumatology patients have flourished in literature, and all the studies have identified an optimal disease control at conception as the main predictor of an uncomplicated gestational outcome in women with different rheumatologic conditions. Even though the care of women willing to become mothers has greatly improved, a further refinement of the process of risk stratification for obstetric complications in patients with rheumatologic conditions is still warranted (Tincani et al., 2019). Given its physiologic role in orchestrating pregnancy progression and its involvement as pathogenic effector in several rheumatologic conditions, complement system is an attractive candidate biomarker to stratify the obstetric risk among women with rheumatologic conditions. Complement testing during gestation is routinely performed in two rheumatic conditions, namely systemic lupus erythematosus (SLE) and anti-phospholipid syndrome (APS). In both diseases, the contribution of complement activation to disease pathogenesis has been extensively documented in animal models. In particular, *in vivo* data have clearly demonstrated that the activation of both the classical and the alternative pathways is required to mediate thrombotic events and pregnancy loss induced by stimulation with anti-phospholipid antibodies (aPL). As recently reviewed, the relevance of activated complement in relation to obstetric complications has been confirmed in SLE as well as APS women: immunohistochemical analysis of placental samples from complicated pregnancies has shown deposition of complement fragments, in particular C4d which provides a fingerprint of complement tissue activation due to its high stability (Chighizola et al., 2020). Furthermore, pregnant women with SLE and APS display higher serum levels of complement degradation products compared to healthy expectant mothers (Kim et al., 2018; Scambi et al., 2019). Despite the progressively raising bulk of evidence, available data on C3 and C4 as predictors of adverse obstetric outcome in lupus and APS are not consistent, not yet allowing to draw definite conclusions about the clinical relevance of complement monitoring throughout pregnancy progression in these settings (Chighizola et al., 2020; Nalli et al., 2021). Of note, in SLE even the association between complement levels during pregnancy and disease flare is questioned, due to the physiologic fluctuation of complement levels during gestation. Nevertheless, it is common practice to monitor complement levels to discriminate between nephritis and pre-eclampsia in SLE expecting mothers presenting new-onset proteinuria: the serum C3 and C4 levels rise in patients with pre-eclampsia, while disease flares are classically characterized by consumed C3 and C4 and raise of anti-dsDNA titres (Mok, 2001).

Scarce evidence on the relevance of complement testing to monitor disease activity and to predict obstetric outcome is available for pregnant patients with rheumatologic conditions other than SLE and APS. However, more and more often these patients express their desire for motherhood to the attending rheumatologist: most commonly, women with inflammatory arthritides, including rheumatoid arthritis (RA) and psoriatic arthritis (PsA); less frequently, women with Sjögren syndrome (SjS) and systemic sclerosis (SSc), which tend to manifest later in life; seldom patient with vasculitides and idiopathic inflammatory myopathies (IIM), which are uncommon conditions.

After addressing the contribution of complement system to the progression of healthy pregnancy and the detrimental role of complement deregulation in pregnancy complications, this review will focus on the relevance of complement in rheumatologic conditions other than SLE and APS, discussing available evidence about the involvement of complement in disease pathogenesis and about the clinical significance of complement serum levels. We will also recapitulate available data on the pregnancy outcome of women with different rheumatologic conditions, in order to clarify the potential role of complement as obstetric surrogate biomarker in these clinical scenarios.

### The Activation of the Complement System

**FIGURE 1:**
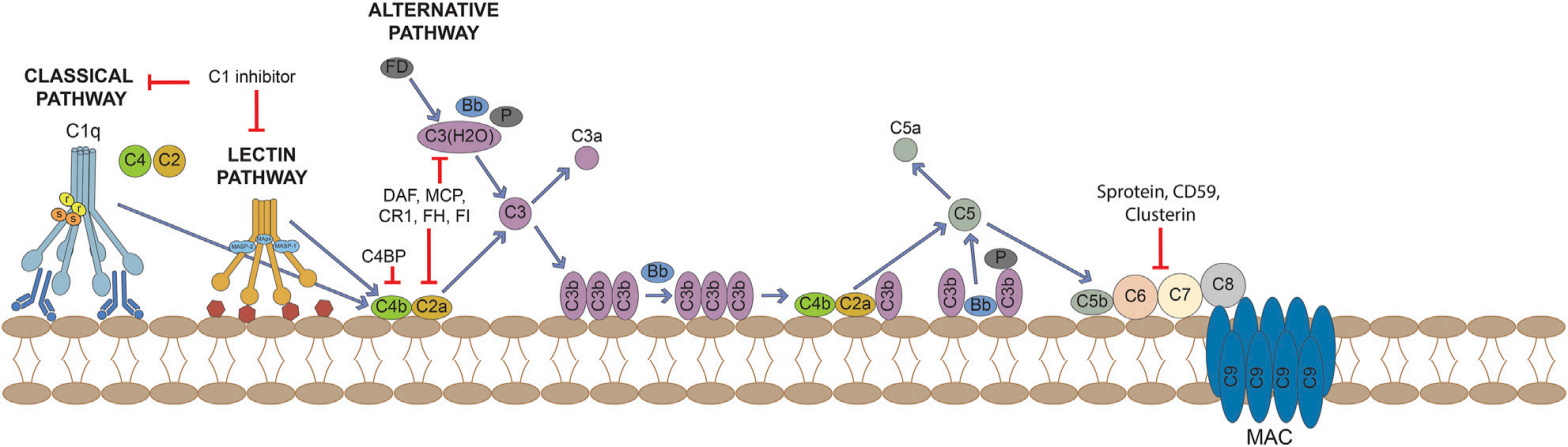
Activation of the complement cascade through the classical, lectin and alternative pathways and its control by regulators and inhibitors.

The complement system is a highly conserved constituent of the innate immunity response and it orchestrates not only the elimination of pathogens, but also immunological and inflammatory processes. This innate surveillance system mediates the clearance of infectious agents by: *1*) opsonization of pathogens, mainly mediated by C3b; *2*) *in situ* recruitment of immune cells, promoted by anaphylotoxins such as C5a and *3*) direct damage to pathogens, thanks to the C5b-C9 membrane attack complex (MAC), also known as terminal complement complex (TCC). Furthermore, the complement system plays an important role in bridging innate and adaptive immunity, as the activation of complement is critical for the development of T cell immunity (Ballanti et al., 2013). The complement system includes more than 60 proteins; the bulk of soluble proteins is produced mainly by the liver, but there is growing evidence that complement proteins can be secreted also locally, playing an important role in regulating physiological complement activation (Lubbers et al., 2017). The activation of complement system can be amplified *via* three different pathways: the classical pathway, the lectin pathway and the alternative pathway (Figure 1). All three pathways culminate in a common terminal cascade, which includes the cleavage of C3 and the deposition of C3b, ultimately resulting in the formation of the MAC and the release of the anaphylotoxins C3a and C5a. MAC forms a membrane pore that inserts into cell membrane and leads to cell lysis; it exists also in a soluble form (sC5b-9), detectable in peripheral blood, which can initiate cytokine synthesis and induce vascular leakage. Very recently, MAC-induced cell activation has been shown to result into a pro-inflammatory cellular profile driven by the activation of the phosphatidylinositol 3-kinase/Akt and ERK signaling pathways, the recruitment of NF-κB and the assembling of the inflammasome, in turn resulting in up-regulation of IL-1β and IL-18 (Xie et al., 2020).

Upon binding to their receptors C3aR and C5aR/CD88, the anaphylotoxins facilitate pathogen clearance by inducing permeability, inflammatory cells chemotaxis and cytokine release. The classical pathway is activated upon C1q binding to immunoglobulins and pentraxins (including C reactive protein [CRP]), DNA, phospholipids (PL) or mithocondrial proteins exposed on the surface of dead cells. These observations imply that the complement is also involved in the clearance of apoptotic cells, which lack complement regulatory molecules, or immune complexes. Once bound to its ligands, C1q leads to the engagements of the enzymes C1r and C1s, which initiate complement activity by cleaving C4 and C2, resulting in the formation of the C3-convertase C4b2a. Similarly, the activation of the lectin pathway follows the engagement of mannose-binding lectin (MBL), ficolins or collectins, molecules that belong to the wide family of pattern-recognition receptors (PPRs), germline-encoded host sensors deputed to the detection of pathogens’ molecules and ligands expressed on self’s death cells. After ligand binding, these recognition molecules activate the MBL-associated serine proteases (MASPs), which can either recruit C4 and C2, ultimately resulting in the formation of the C3-convertase (C4b2a), or directly cleave and activate C3. Thus, the classical and lectin pathways share C2 and C4 complement components in assembly of the C3 convertase. The alternative pathway encompasses a different mechanism of activation, being spontaneously activated upon hydrolysis of C3: C3(H_2_O) is bound by factor B and becomes a target for the cleavage of factor B mediated by the serine-protease factor D. The so formed complex allows the further cleavage of C3 molecules into C3a and C3b. The latter binds to factor B, the subsequent cleavage of bound factor B by factor D results in the assembly of the C3-convertase C3bBb, whose stability is increased by the enzyme properdin. This process is constantly counterbalanced on cell surfaces by the endogenous complement inhibitor factor H, which binds polyanionic residues like sialic acid groups and glycosaminoglycans on human cells but not on pathogens. The alternative pathway acts as an amplification loop, since it can be initiated by C3b formed by any of the three pathways.

The complement system usually operates at a steady state level of activation, and the complement split product C3b is continually deposited on all surfaces of the body that are in contact with plasma. The activation of the complement system is tightly controlled in the fluid phase as well as in tissues, in order to limit both temporally and spatially its proinflammatory and cytotoxic consequences. Some inhibitors bind complement proteins and prevent their incorporation into complement complexes, others bind and terminally inactivate enzymes, other inhibitors serve as a co-factor for enzymatic degradation of complement proteins while other complement inhibitors enhance the decay of C3-convertases. The main regulators of complement activation include membrane-bound regulator proteins CD46/MCP, CD55 and CD59. CD55, also known as decay accelerating factor (DAF), both inhibits C3-convertase formation and accelerates the decay of preformed C3 convertases, whereas MCP facilitates the cleavage of C3b and C4b to their inactive forms. CD59 acts further downstream on the complement cascade, preventing the assembly of the MAC. Soluble complement regulators include C1 inhibitor, C4b-binding protein (C4bp), factors H and I, clusterin and S protein (vitronectin); these regulators restrict the action of complement in body fluids at multiple sites of the cascade reaction (Gialeli et al., 2018).

### Complement Testing and Its Pitfalls

The analysis of fluid-phase complement can be pursued exploiting different approaches: there are assays that allow monitoring the levels of complement proteins, the functional activity of complement or the activation of the complement system *in vivo*. In most laboratories, routine complement evaluation is limited to the immunochemical determination of C3 and C4. These individual complement components are more commonly measured by immunoprecipitation tests (radial immunodiffusion [RID] or nephelometric techniques), even though more sensitive tests such as ELISA and Western blotting are available. However, results are not easy to interpret: hypocomplementemia might result from a genetic defect, which is quite common even among SLE patients. In addition, plasma levels of complement proteins depend on the anabolism as well as catabolism: complement components are acute phase proteins, therefore their degradation might be compensated by accelerated production. In conditions characterized by increased levels of interleukin (IL)-6, such as RA, the excessive production of complement proteins due to inflammation might mask their consumption.

The functional activity of complement pathways is often assessed using haemolytic assays. These techniques unveil whether complement pathways are intact, and abnormal results may drive further testing. They are most used to screen for complement deficiencies but, in expert laboratories, can also be used to evaluate complement consumption. Classical pathway activity (C1, C4 and C2) is commonly measured using the CH50 test, in which serum can be used to lyse sheep erythrocytes coated with anti-sheep antibodies, and the degree of haemolysis is measured. Conversely, the AH50 is the best screening test to evaluate the functionality of the alternative pathway. When both pathways are affected, a deficiency in C3, C5, C6, C7, C8, or C9 should be suspected. The function of complement can also be tested by measuring the deposition of activation products upon activation of the serum with immobilized complement-activating substances (immunoglobulin M [classical pathway], LPS [alternative pathway], or mannose [lectin pathway]) on a microtiter plate. Recently, ELISA-based assays for the measurements of the functional capacities of the three complement pathways have been validated, demonstrating high stability and reproducibility (Palarasah et al., 2011).

The measurement of complement degradation products may be a more sensitive marker of complement activation. Complement activation products are usually present in only trace amounts *in vivo*, but they are rapidly generated *in vitro*. Therefore, it is crucial that samples are collected and stored properly to avoid *in vitro* activation. Activation products to be measured may be either split fragments after enzymatic cleavage of certain components (e.g., C4 [C4a and C4d], C3 [C3a and C3d], factor B [Bb], and C5 [C5a]), or protein complexes, (e.g., C1rs-C1 inhibitor, the properdin-containing alternative pathway convertase C3bBbP, and SC5b-9), where activated components are bound to their respective regulators. Activated split products are measured in plasma (EDTA-anticoagulated blood) due to the interference of the coagulation system enzymes leading to erroneous results. In the last decades, ELISA tests exploiting monoclonal antibodies have replaced techniques as counterimmunoelectrophoresis and crossed immunoelectrophoresis. The main challenges in the measurement of products of activation by ELISA concern the lack of validated assays and the specificity of antibodies. Neoepitope-specific assays, targeting only neoepitopes located exclusively on processed complement proteins and not epitopes on ubiquitous, native molecules, have been described and are partly commercially available for the classical pathway (C1rs-C1 inhibitor, C4d, and C4bc), the alternative pathway (Ba, Bb, and C3bBbP), C3 (C3a, iC3b/C3bc, and C3d) and the terminal reaction sequence (C5a and SC5b-9). The amount of an activation product should be related to the concentration of the native component, since a low level of native component would yield smaller amounts of activation products during *in vivo* activation. Thus, the ratio between the activation product and the native component has been proposed as a more sensitive indicator of *in vivo* activation than the activation product alone (Trouw et al., 2017). Flow citometry allows the detection of cell-bound activation products, mainly erythrocyte-associated. Unfortunately, results are not rapidly available, and reflect complement activation over the 120-day lifespan of the erythrocyte rather than the immediate clinical situation.

Many of the neoepitope-specific antibodies can also be used to detect *in situ* complement activation by immunohistochemistry, but for many conditions tissue biopsy is not an option (Kirschfink and Mollnes 2003). The analysis of complement activity in sequestered tissues, such as synovial fluid, might be helpful, given the local production of complement factors and regulators. In recent years, promising techniques to assess complement activation within particular tissues have been introduced: non-invasive detection of complement activation through magnetic resonance imaging (MRI)-based method has been proposed (Ballanti et al., 2013).

### Complement in Healthy Pregnancy

**FIGURE 2:**
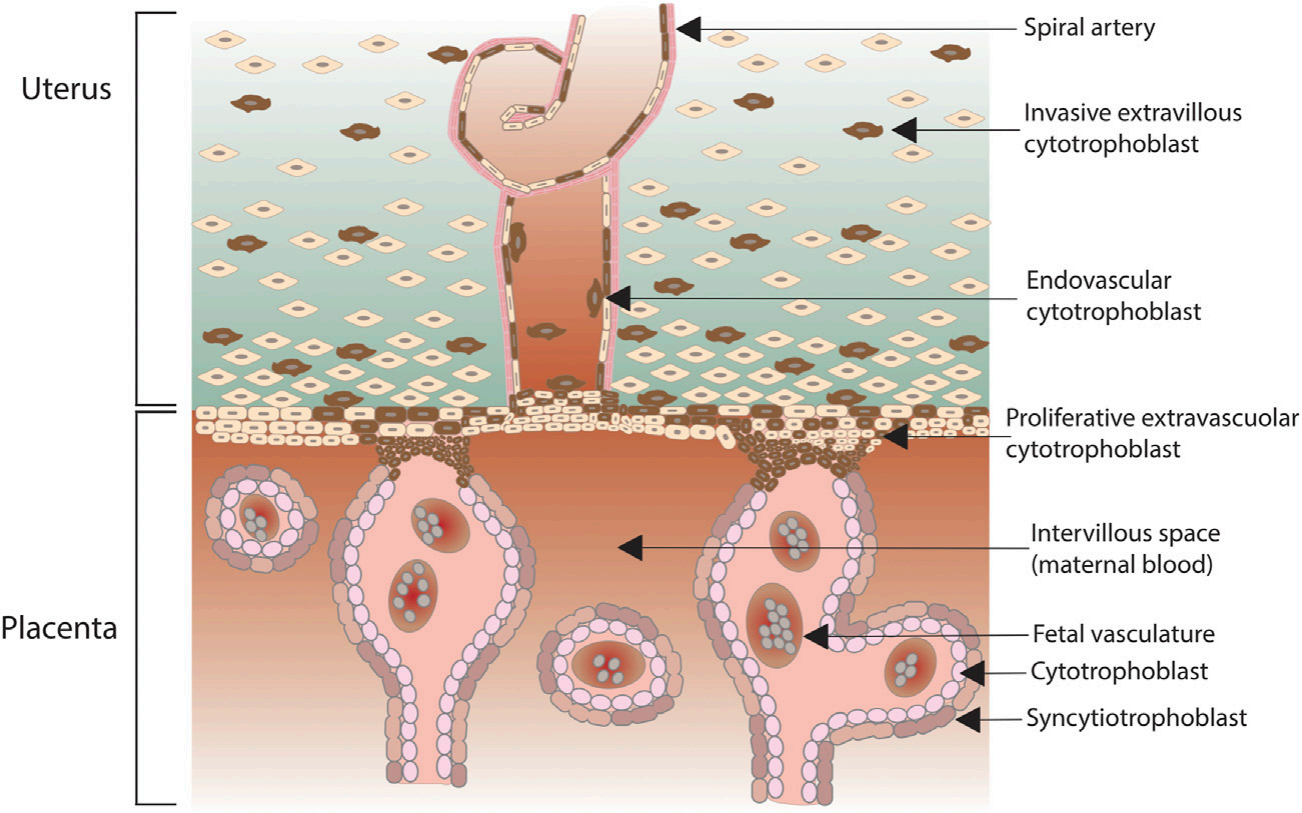
Human placenta, with anchoring villi attached to maternal decidua. Extravillous trophoblasts depart from the villi and invade the decidua surrounding the spiral arteries while some cells enter the lumen of the arteries (endovasculat trophoblasts).

Complement role is critical since the very first stages of pregnancy, as early as in the pre-implantation phase. Indeed, complement factors C3b and CD46 have long been known to facilitate sperm-oocyte interactions. More recently, it has emerged that stage four-cell or eight-cell embryos can synthetize complement and regulators to limit its activation (Reichhardt et al., 2019). In particular, at this early stage C3 exerts a strong embryotrophic potential, *via* conversion to C3b or iC3b: mice lacking C3 have smaller blastocysts (Girardi et al., 2020). The fine-tuning of complement is essential as pregnancy progresses, given that both the placenta and the fetus express paternal antigens, being comparable to a semi-allogeneic graft. In humans, the placenta is a hemochorial structure where maternal blood gets in direct contact with the fetal chorion, the outermost fetal membrane. In particular, placental villi are the fetal structures that connect the extravillous uterus to the fetal chorion (Figure 2). Fetal villi carry the embryo vasculature, and the core of villi contain mesenchymal cells and placental blood vessels, surrounded by two layers of trophoblast, specialized cells of the placenta that exert an important role in embryo implantation and interaction with the decidualised maternal uterus. The trophoblast consists of an inner single layer of mononuclear cytotrophoblast and an outer overlying multinucleated syncytiotrophoblast layer that covers the entire surface of the placenta. The syncytiotrophoblast is in direct contact with the maternal blood that reaches the placental surface. Cytotrophoblasts can further differentiate into extravillous trophoblast, which from the placenta penetrates into the decidualised uterus (Shamonki et al., 2007; Regal et al., 2015). At the syncytiotrophoblast level, the exposition to paternal antigens can induce the activation of the maternal complement system, and the relative hypoxic environment of the normal placenta, usually a driver for trophoblast differentiation, might also act as a trigger for the initiation of the complement cascade. The system must be finely tuned in order to avoid harmful consequences for the fetus: cytotrophoblasts and syncytiotrophoblasts highly express complement regulatory proteins such as DAF, MCP, and CD59. The expression of regulatory proteins decreases with an outer-inner gradient, being minimal in the villous cytotrophoblast, the compartment with the least exposure to maternal blood. However, complement activation is not only critical in maintaining host defense to protect the fetus from infection, but is also required for placental development and fetal survival, with C1q playing a particularly prominent role, as shown by animal models (Girardi et al., 2020). Thus, it is not surprising that complement components can also be synthetized locally: endothelial cells usually do not synthetize complement, but endothelial cells in decidual tissue have been found to secrete C1q and C4; primary throphoblast cells secrete C3 and C4 and express mRNA for components C6, C7, C8 and C9. Staining of term placentae documents the deposition of C3, C3d, C4bp and factor H, with C1q occasionally found in walls of large blood vessels; the TCC C5b-9 has been observed in normal term placenta in the decidua and in the stroma of chorionic villi. In normal uteroplacental spiral arteries, C1q, C3d, C4, C6 and C9 are all evident (Regal et al., 2015).

The plasma levels of complement proteins are well known to increase during gestational course. During physiologic pregnancy, women at 36–37 weeks present increased levels of C4, C4d, C3a, sC5b-9, C3, C9 and factor H compared to non-pregnant controls. The increase in C4d/C4 ratio, the C3a/C3 ratio and sC5b-9 indicates complement activation *via* the classical or lectin pathway in normal pregnancy, without any significant change in the alternative pathway fragment Bb (Derzsy et al., 2010). Accordingly, MBL levels are markedly increased in healthy pregnant women (van de Geijn et al., 2007). The serum levels of C1q remain relatively stable throughout normal pregnancy, which may help to maintain moderate activation of the classical pathway in normal pregnancy (Jia et al., 2019).

### Complement and Adverse Pregnancy Outcome

There is increasing evidence that complement is required for a successful pregnancy, and alterations in the system have been reported in several obstetric complications: early pregnancy loss, pre-eclampsia and pre-term birth. Support to the relevance of complement in recurrent miscarriages comes from animal models: mice lacking C3, C5 and C1q have complicated or non-viable pregnancies. In particular, C5a plays a key role: this anaphylotoxin induces expression of the soluble vascular endothelial growth factor receptor (sVEGFR) ultimately resulting in impaired angiogenesis and adverse pregnancy outcomes (Girardi, 2018). A role in mediating abortion has been advocated even for anti-C1q antibodies: treating pregnant animals with anti-C1q monoclonal antibody induces pregnancy loss, and both the prevalence and titres of anti-C1q antibodies are significantly higher in women with unexplained recurrent pregnancy loss than in healthy parous individuals (Ohmura et al., 2019). Data on circulating levels of complement factors in women with early pregnancy loss are conflicting: one study has identified increased C3 and C4 serum levels as a predictor of subsequent loss (Sugiura-Ogasawara et al., 2006), while other authors have revealed hypocomplementemia in women with recurrent miscarriages (Micheloud et al., 2007). A polymorphism in factor H was found to be protective for recurrent pregnancy loss (Cho et al., 2020). After spontaneous abortion, a significant decrease in complement regulators CD46 and CD55 with an increase in complement activation has been described in the human placenta, reaffirming the importance of limiting placental complement activation to ensure a successful pregnancy (Banadakoppa et al., 2014). Immunohistochemical analysis has revealed C4d deposition in placentae from women with recurrent miscarriages (Girardi, 2018). It has been estimated that approximately 20% of otherwise unexplained early pregnancy loss are due to hypocomplementemia (Girardi, 2018).

Pre-eclampsia consists of the development during pregnancy of hypertension and proteinuria; dysregulated angiogenesis is believed to be implicated in the pathogenesis of the disease, as documented by elevated circulating levels of sVEGFR-1 and of vascular endothelial growth factor receptor 1 (sFlt-1). Animal models suggest that complement, and in particular C1q, is required for a physiologic placentation and for the prevention of pre-eclampsia. Indeed, pregnant C1q-deficient mice develop pre-eclampsia and recapitulate the key features of the human disease: hypertension, albuminuria, endothelial dysfunction, decreased placental VEGF, and elevated levels of sFlt-1, a decoy VEGF receptor (Singh et al., 2011). It has been postulated that complement activation following placental ischemia might induce hypertension and impair foetal growth *via* the endothelin pathway (Regal et al., 2019). Consistently with *in vivo* data, women with previous pre-eclampsia are twice more likely to carry deficiency in C4A and C4B (Regal et al., 2019). Women with pre-eclampsia display an increase in C5a, C3a/C3 ratio in both blood and urine samples; an elevated complement fragment Bb at earlier stages of pregnancy predicts the development of pre-eclamptic manifestations (Denny et al., 2013). Data on sC5b-9 in the plasma of pre-eclamptic women are conflicting, while there is consensus that urinary sC5b-9 is higher in women with severe pre-eclampsia (Denny et al., 2013; Burwick et al., 2018). Plasma levels of complement C1q and factor H are decreased in women with severe pre-eclampsia, both in early-onset and late-onset forms (Derzsy et al., 2010; Agostinis et al., 2016; Jia et al., 2019). Increased MBL levels have been associated with severe (or recurrent) pre-eclampsia in healthy individuals, as well as with additional pregnancy outcomes such as preterm birth, low neonatal birth weight, recurrent miscarriages, and chorioamnionitis (van de Geijn et al., 2007; Than et al., 2008; van de Geijn et al., 2008; Christiansen et al., 2009; Pillay et al., 2019). Higher mRNA amounts of the complement regulator proteins CD55 and CD59, but not CD46, have been found in placental specimens from pre-eclamptic women, a finding that has been interpreted as a compensatory attempt to limit complement activation (Lokki et al., 2014). Complement split products, most notably C4d, have been identified in focal or diffuse staining patterns in the placentae from pre-eclamptic women, in particular in the syncitiotrophoblast (Buurma et al., 2012); lastly, C4d and MAC expression levels in the placental tissue are strongly correlated with sFLT1 levels among pre-eclamptic women (Yonekura Collier et al., 2019).

Similarly, available evidence suggests that activation of the complement system before 20 weeks of gestation independently predicts spontaneous preterm birth: women with elevated circulating levels of the complement factor Bb, a marker of alternative pathway activation, have been found to be 4.3 times more likely to experience a preterm delivery; similarly, elevated plasma levels of C3a have resulted associated with preterm birth (Denny et al., 2013). According to an *ex vivo* study, the interaction of C5a with C5aR might be involved in the cervical remodeling that precedes preterm labour, through engagement of NFkB. NFkB inhibition similarly dampened the release of labour stimulating pro-inflammatory mediators (Lappas et al., 2012). Interestingly, progesterone is thought to prevent preterm labour *via* inhibition of C5a, which acts as an uterotonic in *in vitro* human myometrium (Gonzalez et al., 2014; Romero et al., 2018). However, at the present time it is not clear whether complement activation exerts a causative role in preterm birth, or rather it is an epiphenomenon due to concurrent infection, or a consequence of labour (Denny et al., 2013).

### Complement and Rheumatoid Arthritis

RA is a chronic inflammatory disease that primarily affects the joint but might present with systemic extra-articular manifestations, such as cutaneous vasculitis, serositis and interstitial lung disease. The synovium is the main target in RA; synovitis follows leukocyte infiltration in the joint compartment, an accumulation that is orchestrated by pro-inflammatory cytokines, most notably tumour necrosis factor (TNF)-α and IL-6 (McInnes and Schett, 2011). Antibodies against citrullinated proteins (ACPA) are the serum biomarkers of RA and identify patients with a more aggressive disease course. Even though not sufficient to trigger synovial inflammation, ACPA contribute to aetiopathogenesis by stimulating the production of proinflammatory cytokine production, inducing osteoclastogenesis and promoting NETosis (Kurowska et al., 2017). Furthermore, ACPA were demonstrated to activate complement *via* the classical and the alternative pathways in a dose-dependent manner (Trouw et al., 2009). Several additional triggers have been suggested as potentially involved in complement activation in RA: immune complexes containing rheumatoid factor (RF), fibromodulin and osteoadherin, two small leucine repeat proteins released upon cartilage degradation by matrix metalloproteinases that interact with C1q globular head and CRP (Sjöberg et al., 2005). In chronically inflamed rheumatoid joints, several cells have been shown to contribute to the synthesis of complement factors and receptors: fibroblasts, mononuclear phagocytes, endothelial cells and the lining cells (Whaley et al., 1992). Complement polymorphisms have been shown to be important in genome-wide studies of RA patients; a C5 polymorphism functionally resulted in more efficient cleavage into the proinflammatory anaphylotoxin C5a (Korczowska, 2014).

The different *in vivo* models of RA have concordantly shown that mice deficient in complement components and related proteins (C3, factor B, C5 C5aR, and MASP1/3) are protected against the development of arthritis, with a greater protective effect for C5 than C3 (Holers and Banda, 2018). In particular, the collagen antibody-induced arthritis model suggests that the activation of the alternative pathway of the complement is required for the induction of arthritis in rats. Accordingly, C5 targeting with various methodological approaches results in the amelioration or the resolution of arthritis in animal models. Unfortunately, clinical trials with modulators of complement activation in RA patients have been rather disappointing, with poor clinical efficacy in humans (Holers and Banda, 2018).

Early studies have consistently showed that serum complement levels are normal in RA patients, a finding that might be possibly ascribed to an inflammation-driven increased anabolism or to the local rather than systemic complement activation. Reduced levels of CH50 and/or C4 have been reported in approximately 5% of cases, identifying patients with extra-articular manifestations, most notably rheumatoid vasculitis (Saraux et al., 2001). C1q serum levels have been shown to correlate with clinical disease activity (Holers and Banda, 2018), whereas MBL serum levels have been associated with a favourable outcome (Garred et al., 2000). More recently, RA patients have been found to present higher plasma levels of C2, C3a and sC5b9 compared to control subjects, suggesting a potential activation of the classical pathway (Bemis et al., 2019; Arias de la Rosa et al., 2020). Higher levels of C3b/iC3b, a split product of C3, and factor I were also reported in female compared to male patients, suggesting a counter regulatory mechanisms in women, who are more prone to develop RA (Bemis et al., 2019). Interestingly, among patients with RA, C3 has emerged as marker of metabolic disease: increased C3 was associated with insulin resistance in both obese and non-obese subjects (Ursini et al., 2017a), accurately identifying patients with type 2 diabetes (Ursini and Abenavoli, 2018) and fatty liver disease (Ursini et al., 2017b). Very recently, in a cohort of 200 RA patients, de la Rosa and colleagues reported increased C3 to be associated with cardiometabolic risk and high disease activity at cluster analysis (Arias de la Rosa et al., 2020).

A Brasilian group has investigated the potential modulation of complement activation in response to treatment. A slower hemolytic activity of the alternative but not of the classical pathway was described in patients with inactive or moderately active RA treated with methotrexate (MTX) and infliximab association and, in case of MTX monotherapy, exclusively in those with active RA. Bb levels were increased in MTX-treated patients but not in those receiving also the anti-TNFα agent. C5a levels were found raised in patients with active disease, regardless of treatment (Paoliello-Paschoalato, 2011; Marchi et al., 2018).

The complement system has been investigated even in the synovial fluid of RA patients, with abundant evidence of complement consumption: increased levels of complement factors (C2, C3, C4, C5, MAC) and complement split products (C3a, C3c, C3d, C4d, C5a, sC5b-C9, C1-C1 inhibitor complexes, Ba, Bb) have been described, suggesting a predominant activation of the classical pathway with the alternative pathway being also activated. Immunohistochemistry analysis has demonstrated complement activation also in RA cartilage, with positive staining for C1 and C3b. In inflamed RA synovium, the expression of C2, C3, C4 and of receptors for C3a and C5 has been widely documented, expression of DAF is increased while CD59 expression is reduced (Trouw et al., 2017; Holers and Banda, 2018).

RA is the most common systemic autoimmune disease with a prevalence of 1%, accounting for the large experience that rheumatologists have accumulated in the management of pregnancy in this setting. It is well acknowledged that disease activity during gestational course tends to stabilize or even improve, thanks to pregnancy-related immunomodulation (Förger and Villiger, 2020). Histological and immunofluorescence data on RA placentae are scarce, being limited to the analysis of a single placental specimen from a RA aPL-positive woman, who had a stillbirth. Immunofluorescence evinced the deposition of fibrinogen and IgG, but not complement; it should be underlined that this provides a non specific finding that can be identified even in placentae from healthy women (Ackerman et al., 1999).

A single study has focused on complement in RA pregnancies, in particular analyzing the association of MBL with disease activity and obstetric outcome. The potential relevance of this aspect follows the dual role of MBL in the pathogenesis of RA: on one side, MBL can play a proinflammatory role activating the complement system, on the other side, MBL might exert an anti-inflammatory action by clearing pathogenic agalactosyl IgG immune complexes. Accordingly, in patients with RA, levels of agalactosyl IgG declined during pregnancy, and hence galactosylation increased simultaneously with improvement of RA disease activity. Despite these premises, the authors did not report any association between the MBL genotype groups and changes in RA disease activity or changes in IgG galactosylation during pregnancy and in the postpartum period. Furthermore, MBL genotype groups were not related to the studied pregnancy outcome measures (van de Geijn et al., 2011).

### Complement and Psoriatic Arthritis

PsA is a chronic inflammatory arthropathy that can manifest in approximately 30% of patients with psoriasis. Inflammation mainly targets entheses or the spine: genetic predisposition, microbiome dysregulation and mechanical stress all contribute to increased circulating IL-23 levels, thus favouring the activation of entheses-resident T-cells. The resulting inflammation, mainly IL-22 and IL-17 driven, leads to bone erosion and, eventually, neo-apposition of bone tissue (Ritchlin et al., 2017). There is a wide amount of data about the relevance of complement in psoriasis, while lesser evidence is available for PsA. Serum C3 and C4 levels have been shown to be higher in patients with PsA compared to healthy controls (Chimenti et al., 2012; Ursini et al., 2014; Arias de la Rosa et al., 2020). C3 and C4 levels paralleled disease activity, thus such overexpression is potentially due to concomitant inflammation. C3 has even been proposed as a prognostic marker of response to treatment: in a cohort of 55 PsA patients, higher C3 levels predicted a poorer EULAR response to TNF inhibitors at 22 weeks of follow-up (Chimenti et al., 2012). To investigate the activation of complement cascade, the levels of split products have been assayed in PsA subjects. Increased levels of complement fragments C3b, C4d and Bb have been observed in plasma samples from patients with psoriasis (some with a concomitant arthritis) compared to healthy controls (Rosenberg et al., 1990). Patients with active disease displayed higher levels of sC5b-9, which inversely correlated with the expression of erythrocyte membrane-anchored CD59, an inhibitor of MAC (Triolo et al., 2003). Back in the 90s, it has been hypothesized that complement might be activated by circulating immune complexes even in PsA; however, the expression on erythrocytes of C3b/C4b receptor (CR1), which plays a critical role in the clearance of immune complexes, was not dissimilar in PsA patients and controls (Rivas et al., 1994). Complement activation in PsA has also been investigated at the joint level: in the synovial fluid from PsA patients, C3 levels were higher compared to samples from RA subjects and those with osteoarthritis, even though C3c split product was lower in PsA than RA (Partsch et al., 1991).

Among inflammatory arthritides, PsA is burdened by the highest rate of cardiovascular morbidity and mortality, with a prevalence of metabolic syndrome as high as 40%. Metabolic syndrome is believed to affect cardiovascular risk mainly through insulin resistance, defined as a decreased responsiveness to insulin. Interestingly, in PsA patients serum C3 was the only variable to predict insulin resistance (evaluated with a surrogate measure named HOMA-IR) (Ursini et al., 2014) and whole-body insulin sensitivity (Ursini et al., 2016) in multivariate models. The close interplay between C3, insulin resistance and disease activity in PsA has been recently confirmed in the already cited study by de la Rosa and coworkers. Indeed, these authors recruited also 80 PsA patients, which emerged as the inflammatory arthropathy with the highest prevalence of cardiovascular risk factors (Arias de la Rosa et al., 2020).

### Complement and Sjögren Syndrome

SjS is a slowly progressive, chronic inflammatory autoimmune exocrinopathy whose main clinical feature is sicca syndrome. SjS is regarded as an autoimmune epitheliitis: a lymphocytic infiltrate replace the functional epithelium, leading to reduced exocrine secretions. Histopathological examination of salivary glands documents focal lymphocytic sialadenitis, characterized by lymphocyte infiltration and glandular atrophy, without complement deposition (Goules et al., 2017). SjS might present with systemic extra-glandular manifestations, such as arthritis, cutaneous vasculitis, neurological, pulmonary and renal involvement. The latter might present as tubular interstitial nephritis or, more rarely, as membranous nephropathy. Importantly, SjS patients display a 44-fold increase of the risk of developing lymphoma, in particular an extranodal marginal zone B cell lymphoma of mucosa-associated lymphoid tissue (MALT).

In an experimental murine model, proteomics has recently showed a differential expression of C3 and complement factor H, together with Serpin family G member 1 (SERPING1), fibrinogen α and γ, in the saliva of SjS mice compared to controls (Li et al., 2021). Patients diagnosed with SjS can present hypocomplementemia, in rates ranging between 2 and 25% for C3, 10–39.5% for C4 and 14–15% for CH50 (Skopouli et al., 2000; Ioannidis et al., 2002; Theander et al., 2004; Ramos-Casals et al., 2005; Brito-Zerón et al., 2012; Brito-Zerón et al., 2018; Anquetil et al., 2019; Sebastian et al., 2020). Low complement levels, in particular C4, have been identified as an independent predictor of the development of lymphoma, together with several other clinical variables: chronic parotidomegaly, lymphadenopathy, purpura, lymphopoenia, cryoglobulinemia (Anquetil et al., 2019; Kapsogeorgou et al., 2019). Patients with early onset SjS are more prone to display reduced complementemia compared to those presenting after 35 years of age (Anquetil et al., 2019). Low C3 and C4 serum levels have been identified in a recent metaanalysis as being predictive of mortality (Singh A. G. et al., 2015). In addition, among SjS subjects, low C3, together with hypoalbuminemia and anaemia, was found to be associated with renal involvement (Luo et al., 2019). A single study has evaluated the plasma levels of regulatory protein C4bp in SjS, evincing higher levels in patients compared to controls. Interestingly, plasma C4bp correlated positively with acute phase reactants, C4 and C3 levels and the CD4+⁄CD8+ T-cell ratio, being inversely related to IgG levels and global disease activity (Zadura et al., 2009). In subjects with sicca syndrome, low C4 emerged as a significant predictor of progression towards definite SjS: patients with hypocomplementemia were six times more likely of being later diagnosed with SjS (Shiboski et al., 2018).

The activation of complement in SjS has been investigated even by histopathologic studies. In labial salivary glands specimen from patients with primary SjS, no deposition of C1q and C5b-9 could be detected, thus ruling out a local activation of the classical pathway. However, a differential expression of complement regulatory proteins MCP/CD46 and CD59 was shown in SjS specimens compared to non-specific sialadenitis and healthy controls, potentially suggesting an alternative, cell-mediated mechanism of damage (Legatowicz-Koprowska et al., 2020). A single study has evaluated the pattern of complement deposition in renal biopsies from 39 SjS patients with kidney involvement, describing tubulointerstitial C4d deposition in patients with tubulointerstitial nephritis and in those with membranous nephropathy. In the latter case, all patients presented a pattern of glomerular C4d deposition without concomitant C1q deposition, a finding that is suggestive of the local activation of the lectin pathway (Xia et al., 2019). MBL genotypes have been associated with a less severe presentation of SjS (Ramos-Casals et al., 2008).

Even though the disease usually starts after the 5th decade of life, experience with the management of pregnant SjS women has progressively expanded. The outcomes of approximately 450 pregnancies in SjS have been described: women with SjS can have successful pregnancies, although with a higher rate of pre-term delivery (range 1–27% across studies) and lower neonatal birth weight (Gupta and Gupta, 2017). Unfortunately, no data on complement significance in pregnancies can be extrapolated from the literature.

### Complement and Systemic Sclerosis

SSc is a chronic systemic autoimmune disease clinically characterized by a fibrotic derangement that might affect virtually any organ: most commonly the skin and the gastrointestinal tract, the lungs and the heart (Denton and Khanna, 2017). A fibroproliferative vasculopathy also mediates the manifestations of SSc: Raynaud’s phenomenon, pulmonary arterial hypertension and scleroderma renal crisis (SRC) are the main vascular complications of SSc. The immune system is activated, with the production of mutually exclusive scleroderma-specific antibodies: antibodies against centromere (ACA), DNA topoisomerase I (ATA) and RNA polymerase (ARA) are those included in the updated ACR/EULAR classification criteria for SSc (van den Hoogen et al., 2013).

Currently, the measurement of serum C3 and C4 is not part of routine assessment of scleroderma patients. Following the first description of hypocomplementemia in SSc back in 1967 (Townes, 1967), available data are concordant in reporting an insignificant prevalence of low serum complement levels in SSc ranging from 5.2 to 16.5% across studies (Della Rossa et al., 2001; Cuomo et al., 2008; Foocharoen et al., 2012; Hudson et al., 2014). In the large EUSTAR cohort of 2,540, hypocomplementemia at diagnosis was not associated with any clinical parameter (Foocharoen et al., 2012). A longitudinal study found that hypocomplementemia occurred at least once in 23.4% of patients over a mean follow-up period of 3.4 years (Esposito et al., 2016). Complement levels are reduced more frequently in those with overlapping conditions, most commonly myositis and vasculitis (Hudson et al., 2014; Esposito et al., 2016). Accordingly, low serum complement was associated with autoantibodies commonly found in autoimmune diseases other than SSc: anti-chromatin antibodies (Hudson et al., 2014), anti-ribonucleoprotein antibodies, anti-Ro, anti-Sm and aPL (Esposito et al., 2016).

The scarce studies investigating degradation products documented higher levels of C3d, C3bBbP, C4, C4d and Ba among SSc patients compared to controls (Senaldi et al., 1989; Arason et al., 2002; Okrój et al., 2016), whereas conflicting results are available for plasma C5b-9 (Scambi et al., 2010; Scambi et al., 2015; Okrój et al., 2016). In early times, it was postulated that complement split products might be a surrogate biomarker for diffuse disease subset (Senaldi et al., 1989), an hypothesis that has not been confirmed by following studies (Arason et al., 2002; Scambi et al., 2010; Okrój et al., 2016). Exploiting a cutting-the-edge technique evaluating with mass cytometry a “complosome signature” thanks to a panel of autoantibodies, Arbore and coworkers observed a specific perturbations of the complosome in circulating T cells from patients with SSc (Arbore et al., 2020). Research efforts in scleroderma have been devoted even to the lectin activation pathway, given the role of lectins in mediating ischemia-reperfusion injury (Dobó et al., 2014). To date, four studies have evaluated the lectin activation pathway in SSc, evincing several associations with disease manifestations (Akamata et al., 2014; Osthoff et al., 2014; Osthoff et al., 2019; Miyagawa et al., 2017).

The complement system is locally activated in the skin of scleroderma patients. Indeed, at immunohistochemistry, a pattern of activated complement C5b-9 and C5aR could be detected in involved skin samples from SSc patients, with a perivascular localization (Sprott et al., 2000; Scambi et al., 2015). Consistently, a decreased expression of MCP and DAF was observed at endothelial level, in both affected and non-affected scleroderma skin as compared to cutaneous specimens from healthy subjects or subjects with other connective tissue diseases (Venneker et al., 1994; Scambi et al., 2015). Interesting histopathologic data have been raised in SRC, a life-threatening complication of SSc characterized by the abrupt onset of hypertension, thrombotic microangiopathy and renal insufficiency (Denton and Khanna, 2017). Complement deposition has been analyzed histologically in renal biopsies from SRC patients, observing higher peritubular capillary deposition of C4d (Batal et al., 2009; Devresse et al., 2016) and C1q (Devresse et al., 2016), in particular in those with worse outcome, and more frequently glomerular deposits of C3b (Okrój et al., 2016). Of note, eculizumab, a C5 blocker, was also used as rescue therapy in one case of severe SRC (Devresse et al., 2016).

SSc has a strong female predominance, and usually manifests towards the end of the reproductive life, in the fifth and sixth decade of life. In early times it was believed that pregnancies in SSc should be considered at high risk, with a burden of maternal morbidity and mortality; subsequent studies have minimized the concern, showing a successful gestational outcome in most women. According to a very recent metaanalysis evaluating 16 studies, pregnancies in SSc women were burdened by a greater risk of miscarriage and a 2.8 times higher chance of gestational hypertension. Cesarean delivery was 2.3 times more frequent among SSc women compared to controls. An increased frequency of IUGR emerged; offspring to SSc mothers were more prone to be born prematurely, and with low birth weight. SSc disease activity remained stable in most patients, and peripheral vascular manifestations tended even to improve. Worsening or new disease manifestations were described in 14.3% of cases during gestation and in 10.5% in the 6 months after delivery (Blagojevic et al., 2020). Women with a previous SRC and pulmonary arterial hypertension are at highest risk for severe hemodynamic complications during pregnancy due to the reduced reserve in the pulmonary arterioles to accommodate the increased blood volume and cardiac output that occurs during pregnancy (Chakravarty, 2010).

Available studies have not reported data on complement levels in pregnant SSc women, and the significance of complement fluctuations during pregnancy in scleroderma women has never been addressed.

### Complement and Systemic Vasculitides

Systemic vasculitides are a heterogeneous group of diseases that share a common elementary lesion, namely an inflammatory damage of the vessel wall causing vascular stenosis or thrombosis (Chimenti et al., 2015). The resulting clinical picture depends on the size and the anatomic site of affected vessels. The 2012 updated nomenclature classifies systemic vasculitides according to the vessel size: small vessel vasculitis, medium vessel vasculitis, large vessel vasculitis and variable vessel vasculitis (Jennette et al., 2013). As most systemic vasculitides are rare conditions and usually present in the fifth to seventh decades without a clear female predominance (Chimenti et al., 2015), the experience on the management of pregnancies in women with systemic vasculitides is scant and limited to the conditions whose onset is more frequently reported during reproductive years: vasculitides associated with anti-neutrophil cytoplasmic antibodies (ANCA), Takayasu arteritis and Behçet disease.

Granulomatosis with polyangiitis (GPA), microscopic polyangiitis (MPA) and eosinophilic granulomatosis with polyangiitis (EGPA) belong to the group of systemic necrotizing ANCA-associated vasculitides (AAV). AAV are characterized by positivity for antibodies against proteinase 3 (PR3) and myeloperoxidase (MPO) with an elective involvement of the kidneys, the lungs and the upper airways. The histological hallmark of AAV kidney involvement is pauci-immune necrotizing crescentic glomerulonephritis with scarce immunoglobulin and complement deposition in the glomeruli. This explains why the role of complement system in the aethiopathogenesis of AAV has been historically considered negligible. In the recent decades, however, the scenario has drastically changed (Chen et al., 2017; Brilland et al., 2020): it has emerged that the activation of primed neutrophils by ANCA (Wirthmueller et al., 1997; Xiao et al., 2002; Xiao et al., 2007) leads to the engagement of the alternative complement pathway with generation of C5a and C3bBbP, ultimately resulting in endothelial damage (Ohlsson et al., 2019). It has been proposed that C5a and its neutrophil C5a receptor might act as an amplificatory loop favouring the deleterious effects of ANCA on target cells. In particular, C5a has been reported to prime neutrophils, inducing translocation of ANCA antigens *via* the recruitment of p38 mitogen-activated protein kinase (p38MAPK), extracellular signal-regulated kinase (ERK), phosphoinositol 3-kinase (PI3K) and protein kinase C (Hao et al., 2012; Hao et al., 2013). Even *in vivo* models support complement as a key player in the pathogenesis of AAV: complement activation by alternative pathway, in particularly C5aR, is required for the onset of glomerulonephritis (Schreiber et al., 2009; Xing et al., 2009; Xiao et al., 2014), and mouse knocked-out for C5 as well as factor B–but not C4- were protected from disease onset (Noone et al., 2018). Notably, a C5 monoclonal antibody attenuated disease severity in a mouse model of anti-MPO induced glomerulonephritis (Huugen et al., 2007). Serum hypocomplementemia has been reported in 4.2–20% of patients diagnosed with AAV. Low complement levels at diagnosis have been reported to correlate with a worse prognosis, mainly in terms of poor renal outcome (Molad et al., 2014; Deshayes et al., 2018; García et al., 2019). A recent metaanalysis considering five studies has recently shown that, when the disease is active, AAV patients display increased plasma levels of C5a, soluble C5b-C9 and Bb compared to quiescent phases, further suggesting the selective involvement of the alternative complement pathway (Moiseev et al., 2020). Complement deposition in AAV renal specimens from patients has been described in a significant percentage of patients (Hilhorst et al., 2017; Scaglioni et al., 2017; García et al., 2019), identifying patients with higher proteinuria, disease activity and kidney damage compared to subjects with typical pauci-immune vasculitis (Fukui et al., 2016). Most recently, avacopan, a C5aR antagonist that–differently from eculizumab- does not impair MAC formation, has been investigated as a novel therapy for AAV in a randomized controlled trial enrolling 331 patients receiving either cyclophosphamide followed by azathioprine, or rituximab. At week 52, avacopan 30 mg twice daily was superior to oral prednisone on a tapering schedule with respect to sustained remission (Jayne et al., 2021).

The literature search revealed 133 pregnancies in women with AAV, evincing a higher rate of pre-term birth and IUGR compared to the general obstetric population (Dalakas, 2011; Gatto et al., 2012; Tuin et al., 2012; Nguyen et al., 2021). Women with active disease during gestation are at highest risk of both fetal and maternal complications (Gatto et al., 2012; Tuin et al., 2012; Fredi et al., 2015; Nguyen et al., 2021). Among patients with systemic vasculitides, women with AAV present the highest rate of relapse: most commonly flares -largely of non severe nature- supervene in the third trimester, invariably in case of active disease at the time of conception (Fredi et al., 2015; Nguyen et al., 2021). Reports describing obstetric outcome in AAV women do not provide data on complement levels, possibly due to the only recent interest devoted to complement system in this setting.

Takayasu arteritis is a granulomatous large vessel vasculitis involving the aorta and its branches, usually manifesting in women in the 3rd decade of life (Russo and Katsicas, 2018). The inflammatory endothelial damage might result in stenotic lesions as well as vascular aneurysms; clinical manifestations are secondary to tissue ischemia. The aethiopathogenesis of Takayasu arteritis is poorly elucidated: the current concept envisages that genetic predisposition, infectious agents and immune activation all concur to disease onset. Anti-endothelial cell antibodies (AECA) have been found in up to 94% of patients with Takayasu arteritis (Nityanand et al., 1997). In an *in vitro* study, AECA were found to exert a complement-dependent cytotoxic effect in endothelial cells, potentially contributing to the perpetuation of vascular damage (Tripathy et al., 2001). In agreement with this hypothesis, increased serum levels of C3 and C1q have been proposed as a marker of active disease (Chen et al., 2021). Available literature on pregnancies in Takayasu arteritis is solid, with approximately 700 described pregnancies. The most common obstetric complications observed in women with Takayasu arteritis relate to severe hypertension and preeclampsia, which are associated with intrauterine fetal death and IUGR. Pregnancy does not seem to impact on disease activity: inflammation even attenuates during gestation, and flares during pregnancy are rare events (Hirayama et al., 2015; Nguyen et al., 2021; Gatto et al., 2012; Fredi et al., 2015; Chen et al., 2021; Hidaka et al., 2012; Tanaka et al., 2014; Alpay-Kanitez et al., 2015; Mandal et al., 2012; Comarmond et al., 2015; de Jesús et al., 2012; Singh N. et al., 2015). The complement system has not been evaluated in pregnant women with Takayasu arteritis.

Behçet disease is a chronic relapsing inflammatory disease characterized by oral and genital ulcers, ocular involvement, gastrointestinal manifestations and thrombosis (Machen and Clowse, 2017). The pathogenic steps leading to Behçet disease are poorly characterized, but it is currently believed that exogenous factors in genetically susceptible individuals are implicated (Bulur and Onder, 2017). Data on the role of the complement system in Behçet disease are still limited, coming from a single Chinese group. These authors reported that the expression of C3aR was increased in peripheral blood mononuclear cells (PBMC) from patients with active Behçet disease and, upon stimulation with serum samples from patients, the expression of C3aR was significantly induced in PBMC (Wang et al., 2017). In addition, these researchers evinced that a high gene copy number of the complement components C3 and C5 and the rs408290 GG genotype of C3 and rs2269067 GG genotype of C5 are risk factors for Beçhet disease (Xu et al., 2015).

Behçet disease often presents during fertile years, therefore pregnancies in women with this condition are often seen in clinical practice. In the literature, there are reports of approximately 550 pregnancies. In the largest series to date published, women with Behçet disease appeared to be at greater risk for preterm labour and postpartum venous thromboembolism (Lee et al., 2019). The rate of obstetric complications in women with Behçet disease is similar to the general population. Disease activity improves in most cases, especially in women treated with colchicine. Less than half of women experience a disease relapse, more commonly arthritis, uveitis and cutaneous manifestations (Gatto et al., 2012; Noel et al., 2013; Iskender et al., 2014; Fredi et al., 2015). None of the available studies evaluated the relevance of complement in pregnant women with Behçet disease.

### Complement and Idiopathic Inflammatory Myopathies

IIM are systemic diseases that share a predominant skeletal muscle involvement but differ in pathogenetic mechanisms. Polymyositis (PM) is regarded as a T-cell mediated disease, while dermatomyositis (DM) has been identified as a complement-mediated microangiopathy (Dalakas, 2011). Indeed, skeletal muscle samples from DM patients demonstrated C5b-9 deposits on endomisial and perimisial arterioles, suggesting a prominent role of complement system in mediating vessel injury, particularly in case of childhood onset (Kissel et al., 1986). Not surprisingly, complement deposits have been proposed as a diagnostic tool in order to distinguish DM from the other IIM (Kissel et al., 1986; Jain et al., 2011). Moreover, eculizumab, an anti-C5, has been proposed as a savage therapy to control disease activity in refractory DM, with 11 successful cases described to date (Takada et al., 2002; Faguer et al., 2018). Nevertheless, two case reports described patients with DM lesions but complement deficiency, namely C2 (Leddy et al., 1974) and C9, the latter preventing MAC formation (Ichikawa et al., 2001). Macrophages are considered as the key effectors in immune mediated necrotizing myositis (IMNM), a disease characterized by muscle fiber necrosis with positivity for antibodies against signal recognition particle (SRP) and 3-hydrixy-3-methylglutarylcoenzyme A (HMGCR). At immunohistochemical analysis, sarcolemmal C5b-9 and C1q deposits have been observed in samples from IMNM patients. These observations suggest that the activation of the classical complement pathway is involved in disease pathogenesis, potentially *via* a mechanism of antibody-dependent cell-mediated cytotoxicity (Drouot et al., 2014; Allenbach et al., 2018). Although serum levels of complement are historically regarded to be normal or increased in IIM, the evidence in support of this belief is poor. Few studies have investigated levels of C3, C4 and complement split products in IIM; also, it is worth noticing that, in the past decades, the poor insights about immunopathologic differences between IIM led to an overdiagnosis of PM over the other subtypes (Bottai et al., 2017). Due to this limitation, some authors reached controversial results: back in early 80s, Behan and coworkers observed reduced C3 and C4 levels respectively in two and nine out of 33 PM patients (Behan et al., 1982); the same author observed a significant lowering of C4 (but not C3) levels associated with a parallel tendency of C1q levels in six out of eight PM patients, but variable levels of Factor B (Behan and Behan, 1977); other authors described serum complement increase in PM (Wedgewood and Janeway, 1953; Williams and Law, 1958). With regard to DM, Scott described elevated C3d levels in six out of seven patients with active juvenile DM (Scott and Arroyave, 1987); Campo and colleagues observed a correlation between serum levels of C3a and, to a lesser extent, C5b-9 with disease activity in 16 patients with DM (Campo et al., 2007); elevated serum C5b-9 levels and higher deposition of activated C3 fragments on red cells (as measured by an *in vitro* C3 uptake assay) were reported in a cohort of 13 DM patients, which normalized after treatment with intravenous immunoglobulins (Basta and Dalakas, 1994). Moreover, Li and coworkers described a positive correlation between serum C1q levels and inflammation in patients with IIM (Li et al., 2019).

Literature on pregnancies in IIM suggests that fetal prognosis reflects the activity of maternal disease (Silva, 2003; Váncsa et al., 2007; Chopra et al., 2008; Nagy-Vincze et al., 2014; Chen et al., 2015). According to available studies, including a large population-based study, in well-controlled patients the risk of complications mainly relates to the onset of hypertensive disorders such as preeclampsia and eclampsia (Chopra et al., 2008; Chen et al., 2015; Kolstad et al., 2018). Vancsa et al. observed 14 pregnancies after the onset of IIM, reporting an increased incidence of IUGR, prematurity and fetal loss only in patients with active disease at conception while women with disease in remission had uneventful pregnancies (Váncsa et al., 2007). The rate of disease flare in women with childhood onset IIM was as high as 40% (Silva, 2003); in those with adult-onset disease, flares occurred mostly in the post-partum (Pinal-Fernandez et al., 2014). A poor fetal outcome has been described in case of disease onset during pregnancy (Silva, 2003). To date, no studies about complement levels in pregnant women with IIM are available.

## Conclusion

The role of complement in the pathogenesis of rheumatologic conditions has been neglected for years, but evidence of its contribution to disease onset has been recently accumulating in many different conditions in the field of rheumatology. In RA, available data suggest that the complement system, mainly the classical pathway, contributes to tissue damage especially in ACPA positive subjects, and that complement activation mostly occurs locally in the joint microenvironment. These observations account for the poor clinical significance of the measurement of the serum complement levels. Data in support of a pathogenic role for complement activation are rather solid also in ANCA-associated vasculitides and DM. In vasculitides, complement plays a dual role: on one hand, ANCA stimulation of neutrophils results in the activation of the alternative pathway, on the other, C5a induces traslocation of ANCA antigens, thus favouring the pathogenic role of antibodies. Histologically, among patients with AAV vasculitides complement deposition in the kidneys identifies patients with more aggressive renal disease, while in DM sC5b-9 deposits are invariably present, classically distributed around endomisial and perimisial arterioles. Data about a potential contribution of complement pathways in PsA are rather dated and poorly consistent, whereas hints about complement involvement in SSc have been accumulated only recently, following the evidence of complement deposition in affected skin and in renal samples from SRC patients. C3 and C1q serum levels have been explored in two studies as potential markers of disease activity in Takayasu arteritis, whereas data in Behçet disease are limited to *in vitro* observations.

Among patients with SjS, hypocomplementemia exerts a prognostic role, as it allows identifying patients at risk of extra-glandular manifestations and predicts mortality. AAV patients with active disease are more likely to display low serum levels of C3 and C4, which thus might be a candidate biomarker in this setting. Despite the current notion of DM as a complement-mediated disease, data on C3 and C4 fluctuation during disease course are scarce and inconsistent. Surely it would be worthwhile for the rheumatology community to devote additional research effort to better capture the contribution of complement activation in the pathogenesis of rheumatic conditions and also to better characterize the significance of monitoring C3 and C4 in clinical practice. Firstly, the recently unravelled mechanisms of MAC-mediated signalling might shed new light on the contribution of complement system to the pathogenesis of rheumatologic diseases (Xie et al., 2020). For instance, NF-kB inducing kinase, a MAC-dependent activator of non canonical NF-kB signaling, has been recently shown to mediate in an *in vivo* model allograft vasculopathy, an immune-mediated vascular remodelling consisting in diffuse stenosis affecting the vasculature of solid organ transplant (Qin et al., 2016). Over the years, the number of rheumatological diseases in which complement was found to play a pivotal role has progressively raised. This is the case even of osteoarthritis, a very common aging-related condition traditionally considered to be pauci inflammatory. In particular, proteins of the classical (C1s and C4A) and alternative (factor B) pathways, the central components C3 and C5, and the C5, C7, and C9 components of MAC were all found to be aberrantly expressed in osteoarthritic compared to healthy synovial fluids. Complement activation emerges in patients in early phases of osteoarthritis as well as those with end-stage disease, and seems to occur locally in the synovial tissue, possibly triggered by extra-cellular matrix components of the cartilage released by or exposed in osteoarthritic tissue. The relevance of complement in osteoarthritis has also been confirmed in animal models: C5-deficient and C6-deficient mice, but not C3-deficient animals, were protected against osteoarthritis induced by medial meniscectomy while mice deficient in CD59a developed a more severe phenotype of osteoarthritis (Gobezie et al., 2007; Wang et al., 2011). Despite the many progresses, the complement system still offers a wealth of surprises to researchers: in the last decade, the link between complement activation, inflammation and cardiometabolism has been corroborated by extensive evidence. Such observation is highly relevant even in the field of rheumatology, given the high cardiovascular burden that patients with rheumatologic conditions still experience. In the general population, increased levels of C3 and C4 have been associated with metabolic syndrome (Xin et al., 2018), a relationship underpinned by the fact that the adipose tissue can synthesize complement factors. An excessive amount of body fat mediates the oversynthesis of complement factors, in particular C3. This results in increased levels of C3a-desArg, a complement fragment that acts as a paracrine metabolic factor stimulating lipogenesis and modulating blood pressure (Vlaicu et al., 2016). Furthermore, local production of complement factors in the adipose tissue might account for the clinically relevant link between obesity and low-grade inflammation (Barbu et al., 2015). However, C3 correlates with insulin secretion independently of adiposity measures in non-diabetic subjects, arguing against the hypothesis that an expansion of fat mass might fully explain this association (Fiorentino et al., 2015). In several cohort studies, increased C3 and C4 correlated well with cardiovascular risk factors, and men with high C3 displayed an increased incidence of cardiovascular events, including stroke and myocardial infarction (Engström et al., 2007b) and were at higher risk of subsequent development of arterial hypertension (Engström et al., 2007a). Such intertwining between complement, cardiovascular risk and inflammation acquires particular relevance in rheumatologic conditions, which are burdened by troublesome rates of metabolic syndrome and cardiovascular events.

Despite the raising pathogenic relevance of complement in rheumatologic conditions and in mediating non autoimmune models of pregnancy complications, complement levels have been scarcely investigated as markers of obstetric outcome in pregnant women with rheumatologic conditions. This is rather surprising, considering that, even in the third millennium, pregnancies in women with rheumatologic conditions are frequently complicated and easily available prognostic biomarkers are highly warranted. As a matter of fact, to date APS is the only condition in which solid experimental data have been raised about the role of complement in mediating obstetric complications. However, the few studies focusing on complement levels in pregnant women with SLE and/or APS have not yet provided a clear-cut evidence of their clinical significance; nevertheless, the current practice considers low C3 and C4 as prognostic marker of obstetric unfavorable outcome.

Surely there is an urgent need of focusing future research on the significance of monitoring complement levels throughout gestation in women with rheumatologic conditions, but data should be adequately collected in order to optimize the interpretation of data and the reliability of conclusions. Indeed, the interpretation of complement levels is extremely complex in pregnant women: the subtle balance between anabolism and catabolism undergoes several physiologic modifications during gestational course, which might bias the accuracy of results. To raise clear-cut evidence in women with rheumatologic conditions, studies should enroll healthy pregnant women, matched for gestational age, and account for potential confounders, such as treatment. In particular, it is important to consider that heparin, one of the most used pharmacological tools in pregnant women with rheumatologic conditions, inhibits complement activation, modulating the alternative pathway at the C3 convertase level (te Velthuis et al., 1996). More recently, heparin and its derivative low molecular weight heparin were shown to interact with C1q during pregnancy (Oberkersch et al., 2010). Treatment with heparin has been confirmed to mitigate complement activation both in animal models and in humans (Zaferani et al., 2014). It should be also highlighted that the ultimate evaluation of the role of complement in mediating pregnancy failure comes from the histopathological evaluation of placenta, which is still lacking in all rheumatologic conditions other than SLE and APS.

In conclusion, complement activation is undoubtedly involved in complicated pregnancies in the general population. Data on complement as prognostic tool in pregnant patients with rheumatologic conditions need to be further explored, identifying an emerging research field and providing a challenge for future studies. Indeed, this scenario might pave the way to unexplored frontiers in terms of disease monitoring and pharmacological approaches, such as the use of complement-targeting therapeutic tools in order to improve obstetric outcome in women with rheumatologic conditions. This is the case of, the humanized anti-C5a antibody eculizumab that strongly binds C5 and interferes with its cleavage to the pro-inflammatory C5a and the terminal complement member C5b-9. Due to its unique IgG2/4κ structure, it has a weakened Fc functionality that limits its passage through the placental barrier and therefore theoretically renders it safe in pregnancy even though long-term data about children exposed *in utero* to eculizumab are yet not available (Beltagy et al., 2021).
